# Pyopneumothorax with bronchopleural fistula due to pulmonary infection caused by *Porphyromonas gingivalis* in a patient with periodontitis

**DOI:** 10.1111/crj.13684

**Published:** 2023-08-13

**Authors:** Jun Sha, Jie Shao, Sheng Lu, Wei Yao, Yimai Deng, Jie Chen, Jianfeng Zhang, Yufeng Feng

**Affiliations:** ^1^ Intensive Care Unit Changshu No.2 People's Hospital Changshu China; ^2^ Changshu Medicine Examination Institute Changshu China

**Keywords:** fistula, infection, periodontitis, *Porphyromonas gingivalis*, Pyopneumothorax with bronchopleural

## Abstract

Pyopneumothorax with bronchopleural fistula is a rare complication of lung infection. We herein report a case of pyopneumothorax with bronchopleural fistula caused by *Porphyromonas gingivalis* infection, a common pathogenic pathogen of periodontitis, in a 49‐year‐old man with periodontitis. The patient was admitted with respiratory failure. Pleural puncture yielded a lot of gas continually and foul‐smelling light brown pus, which was found to be caused due to infection with *P. gingivalis* by the metagenomic next generation sequencing (mNGS) and anaerobic culture.

## Graphical abstract

Pyopneumothorax with bronchopleural fistula is a rare complication of lung infection. We herein report a case of pyopneumothorax with bronchopleural fistula caused by *Porphyromonas gingivalis* infection, a common pathogenic pathogen of periodontitis, in a 49‐year‐old man with periodontitis. The patient was admitted with respiratory failure. Pleural puncture yielded a lot of gas continually and foul‐smelling light brown pus, which was found to be caused due to infection with *P. gingivalis* by the next generation sequencing (NGS) and anaerobic culture.

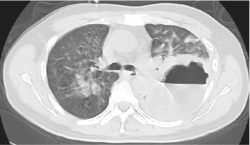

## 1 INTRODUCTION

*Porphyromonas gingivalis* is a widely recognized pathogen leading to periodontitis.^1^ *P. gingivalis* was reportedly found in more than 80% of subgingival plaque in patients with periodontitis.^2^ This bacteria produces a various array of virulence factors, including capsule, lipoplysaccharide, proteolytic enzymes, and fimbriae, and rarely causes extraoral infection, but it once causes extraoral infection, which can lead to severe inflammation, tissue destruction, and sometimes abscess formation.^3^ Pyopneumothorax with bronchopleural fistula is an infrequent complication of pulmonary infection.^4^ Pyopneumothorax with bronchopleural fistula is characterized by the accumulation of pus and gas in the chest cavity. It is commonly seen in severe lung infections that invades the pleural cavity.^5^ To the best of our knowledge, we report the first case of *P. gingivalis* causing pyopneumothorax with bronchopleural fistula.

## 2 CASE REPORT

**FIGURE 1:**
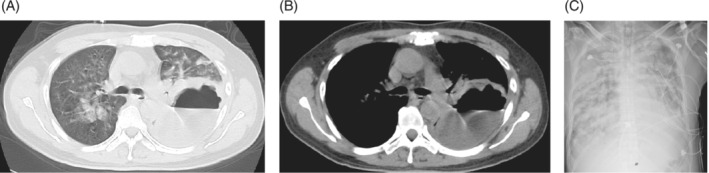
(A) The patient's chest CT lung window on admission. (B) The patient's chest CT mediastinal window on admission. (C) Chest X‐ray of the patient on admission.

A 49‐year‐old man with a history of tobacco and alcohol consumption was admitted to the hospital due to cough and sputum for a week and dyspnea for a day. Physical examination indicated a fever (38°C), shortness of breath with pus odor, audible moist rales in both lungs, and low breath sounds in the left lung. He had coughed and produced phlegm, which was light brown purulent sputum. Blood tests showed increased white blood cells (14 700 cells/μL with 92.8% neutrophils) and C‐reactive protein (285.6 mg/L). A chest computed tomography scan showed infectious lesions in both lungs, the giant cavity of the left lung with gas and fluid levels (Figure 1A–C).

**FIGURE 2:**
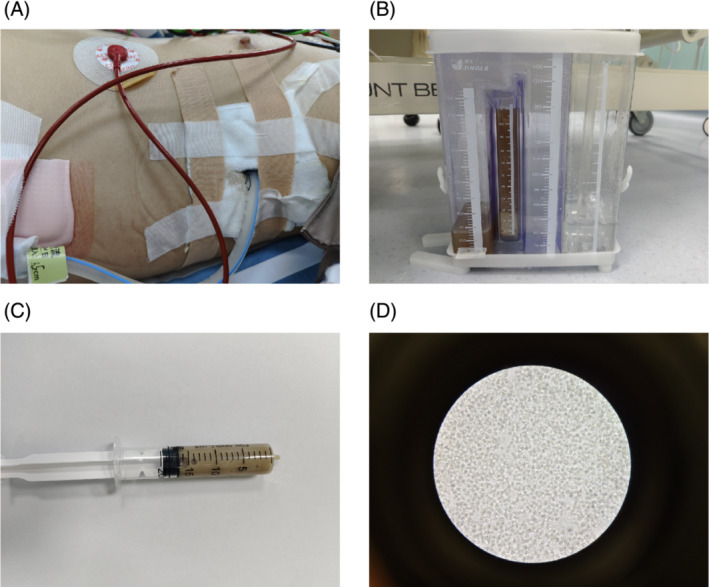
(A) Closed thoracic drainage was performed. (B) A large amount of pale brown pus and a large amount of gas overflowed from the drainage bottle. (C) Pale brown pus drawn from the drain bottle. (D) A large number of pus cells were observed under the microscope after the pus smear.

**FIGURE 3:**
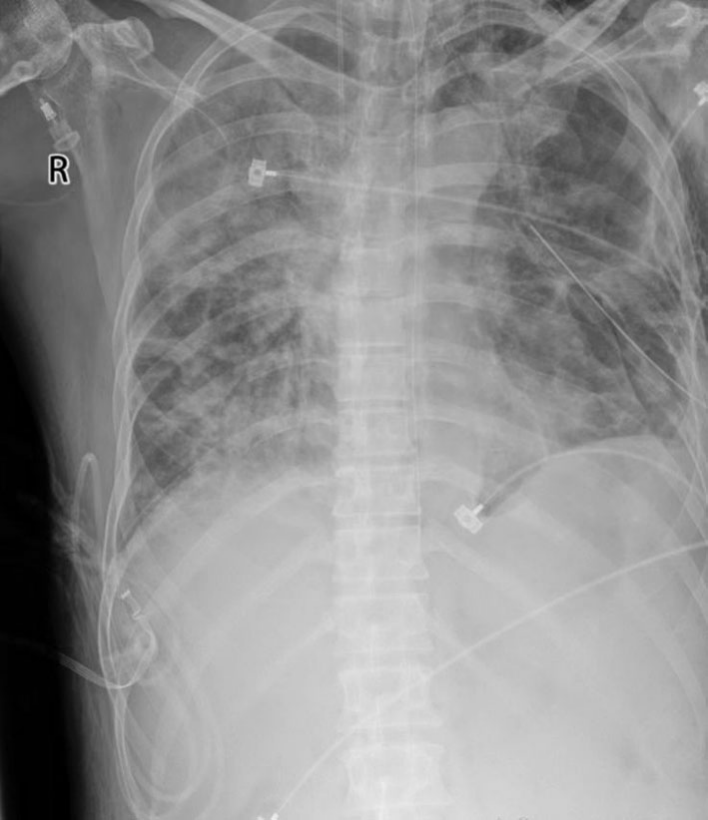
Chest X‐ray taken after treatment with imipenem showed improvements of chest wall mass and lung opacification.

**FIGURE 4:**
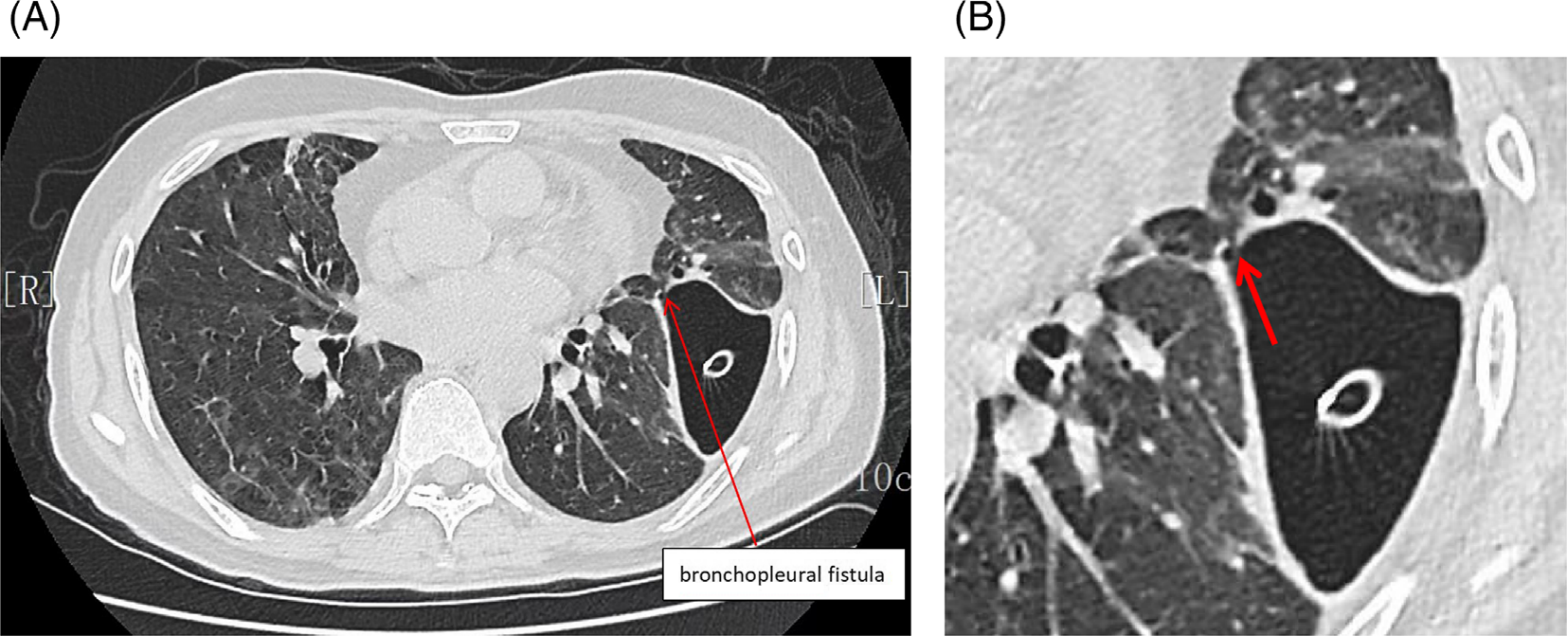
(A) There was a 1.41 mm diameter fistula in the left superior lobe of the lung connecting to the chest cavity. (B) Enlarged chest CT image of a bronchopleural fistula.

The patient developed respiratory failure and underwent mechanical ventilation with endotracheal intubation and closed thoracic drainage, which produced gas continuously and a large amount of light brown pus with a foul odor (Figure 2A–C). Many pus cells were seen under the microscope after taking pus for smear (Figure 2D). Meanwhile, the pus and peripheral blood were tested for NGS. Based on these findings, a diagnosis of pyopneumothorax was made, Gram‐positive suppurative bacterial infection was considered more likely, and intravenous treatment with linezolid was initiated. However, the inflammatory markers continued to rise after 2 days of linezolid anti‐infection. Two days later, the NGS results of pus and peripheral blood indicated *P. gingivalis* positive and *P. gingivalis* abundance was very high. Then the antibiotic was immediately changed to imipenem (0.5 g q6h) according to the bacterial resistance. Meanwhile, Gram staining of the pus was Gram‐negative, and *P. gingivalis* was cultured in the anaerobic culture of the pus a few days later. The patient had an attack of periodontitis a month ago, which was highly suspected to be the source of the *P. gingivalis* lung infection. After anti‐infective treatment with imipenem, the inflammatory index C‐reactive protein and procalcitonin decreased significantly. At the same time, the ventilator support parameters of the patient were reduced, and the blood gas condition improved. The patient's improvement was also evident on the reexamined chest X‐ray (Figure 3). However, a large amount of gas continued to escape from the chest closed drainage bottle, and the bronchopleural fistula was considered according to the chest CT upon admission. Unfortunately, the fistula was not located by chest CT. After the patient's condition improved, the tracheal cannula was successfully removed and replaced with the nasal catheter for oxygen inhalation. After a period of conservative treatment, a review of the chest CT revealed a 1.41 mm diameter fistula in the left superior lobe of the lung connecting to the chest cavity (Figure 4A,B). After nearly 3 months of long‐term life with the tube, no gas escaped from the chest drainage bottle, and the chest tube was successfully removed.

## 3 DISCUSSION

*P. gingivalis* is a kind of Gram‐negative anaerobic bacteria, which is widely regarded as the main pathogenic bacteria causing periodontitis. *P. gingivalis* can enter the lungs by aspiration and cause lung infection. In recent years, more and more studies have found that *P. gingivalis* is closely related not only to periodontitis and oral squamous cell carcinoma^6^ but also to inflammatory bowel disease,^7^ cancer,^8^ cardiovascular disease,^9^ Alzheimer's disease,^10^ rheumatoid arthritis,^11^ diabetes,^12^ and other non‐oral diseases. An external oral abscess caused by *P. gingivalis* is rare, but multiple cases of brain abscess^13^ and one case of lung abscess have been reported. Pyopneumothorax with bronchopleural fistula from *P. gingivalis* is very rare. A case of empyema and subcutaneous abscess caused by *P. gingivalis* infection was reported.^14^ To the best of our knowledge, this case report is the first case of pyopneumothorax with bronchopleural fistula caused by *P. gingivalis*.

Empyema is the accumulation of pus in the pleural cavity caused by a suppurative infection of the pleural cavity. Pathogenic bacteria can spread to the pleural cavity to cause empyema through pulmonary infection, blood flow, lymphatic vessels, thoracic puncture, and other ways, among which pulmonary infection is more common.^15^ For the already formed empyema, treatment with antibiotics alone is easy to cause the relapse of empyema. Effective anti‐infective treatment should be accompanied by adequate drainage of the abscess, which will have a better prognosis.^16^ Pus and peripheral blood should be submitted for bacterial culture or genetic testing before antibiotic application and preferably obtained bacterial culture or genetic test results as soon as possible. However, when patients develop large‐diameter bronchopleural fistula, conservative treatment is usually ineffective and surgical intervention is required.^4^

In conclusion, this case report suggests that *P. gingivalis* can cause severe pulmonary infection and pyopneumothorax with bronchopleural fistula. A recent history of periodontitis is crucial information for clinical treatment.

## AUTHOR CONTRIBUTIONS

**Jun Sha:** Write the article. **Jie Shao, Sheng Lu, Wei Yao, Yimai Deng, Jie Chen, and Jianfeng Zhang:** Case data collation. **Yufeng Feng:** Review and revise the article.

## ACKNOWLEDGEMENTS

This research did not receive any specific grant from funding agencies in the public, commercial, or not‐for‐profit sectors.

## CONFLICT OF INTEREST STATEMENT

The authors declare that there are no conflict of interests. We do not have any possible conflicts of interest.

## ETHICS STATEMENT

The case report had been reviewed by the Ethics Committee of Changshu No.2 People's Hospital.

## Data Availability

The data that support the findings of this study are available from the corresponding author upon reasonable request.
